# Measuring Lactase Enzymatic Activity in the Teaching Lab

**DOI:** 10.3791/54377

**Published:** 2018-08-06

**Authors:** Cattleya S. Leksmono, Claudia Manzoni, James E. Tomkins, Walter Lucchesi, Graeme Cottrell, Patrick A. Lewis

**Affiliations:** ^1^School of Pharmacy, University of Reading; ^2^Department of Molecular Neuroscience, UCL Institute of Neurology; ^3^School of Biological Sciences, Royal Hollway, University of London

**Keywords:** Biology, Issue 138, Lactase, Enzymatic Assay, Lactose, Hydrolysis, Spectrophotometer, Ortho-Nitrophenol-*Beta*-D-galactopyranoside, Enzyme Kinetics

## Abstract

Understanding how enzymes work, and relating this to real life examples, is critical to a wide range of undergraduate degrees in the biological and biomedical sciences. This easy to follow protocol was developed for first year undergraduate pharmacy students and provides an entry-level introduction to enzyme reactions and analytical procedures for enzyme analysis. The enzyme of choice is lactase, as this represents an example of a commercially available enzyme relevant to human disease/pharmaceutical practice. Lactase is extracted from dietary supplement tablets, and assessed using a colorimetric assay based upon hydrolysis of an artificial substrate for lactase (ortho-nitrophenol-*beta*-D-galactopyranoside, ONPG). Release of ortho-nitrophenol following the hydrolytic cleavage of ONPG by lactase is measured by a change in absorbance at 420 nm, and the effect of the temperature on the enzymatic reaction is evaluated by carrying out the reaction on ice, at room temperature and at 37 °C. More advanced analysis can be implemented using this protocol by assessing the enzyme activity under different conditions and using different reagents.

**Figure:**
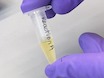


## Introduction

Enzymes are a specialized type of protein that act as biological catalysts for chemical reactions in living organisms^1^. The action of enzymes is critical for life, providing energy, disposing of waste and allowing organisms to function. Understanding enzymes is, therefore, critical for a full understanding of life. Such knowledge is essential for a wide variety of university level degree programs, ranging from the medical sciences to biology. While a detailed background ranging from principles of catalysis to theoretical models of enzyme activity can be provided to students using lectures and reading material, the properties of enzyme reactions are best comprehended by hands on practical experience of enzymes in action as previously demonstrated^2^. This protocol provides a simple to follow experimental paradigm for measuring enzyme activity under laboratory conditions, using lactase as an example of an enzyme with an activity relevant to human nutrition and health.

The glycosidic hydrolase lactase (EC 3.2.1.23/26) is an enzyme of central nutritional importance for mammals^3^. The activity of lactase is highly conserved through evolution, and derives from the beta-galactosidase family of enzymes — a family present from *Escherichia coli* through to* Homo sapiens* (**Figure 1**, PDB 1JZ8)^4^. The critical importance of lactase in a human nutritional setting stems from its role in allowing the breakdown of lactose into its constituent monosaccharide components, which can then be used to generate energy in the body. Lactase catalyzes the hydrolysis of the glycosidic bond in the disaccharide lactose, releasing galactose and glucose (**Figure 2**)^5^. These monosaccharides are then used primarily for the generation of adenosine triphosphate (ATP) *via *the citric acid cycle and oxidative phosphorylation^6^. During neonate and infant development, lactase is highly expressed in the human digestive system, breaking down lactose received from breast milk of which lactose is the primary carbohydrate component, and one of the key sources of nutrition during early years^7^. The medical importance of lactase is highlighted by congenital lactase deficiency (CLD), a rare autosomal recessive condition caused by mutations in the lactase gene (*LCT*) coding for the lactase enzyme^8^. New-born babies with CLD exhibit very little lactase activity, thus they cannot be fed on breast milk, any other type of milk, or formula containing lactose.

During childhood, lactase expression is normally reduced; however, this reduction following weaning varies geographically, with approximately 35% of adults worldwide continuing to express the enzyme^9^. Sustained expression of lactase, known as lactase persistence, allows individuals to continue to digest milk and dairy products from a range of sources. Conversely, the loss of lactase expression can lead to lactose intolerance, also known as adult-type hypolactasia (ATH), resulting from an inability to break down lactose in the gut. ATH is characterized by a build up of lactose in the colon following ingestion of lactose containing food products. In the colon, the accumulated lactose is fermented by gut microbial fauna, releasing gasses including hydrogen, methane, and carbon dioxide. The production of these gases in individuals with lactase enzyme deficiencies promote abdominal bloating, increased flatulence, pain, nausea, and borborygmi (stomach rumbling)^7^. Increased levels of lactose in the digestive tract can also lead to loose stools.

The control of *LCT* gene expression is modulated by polymorphisms located in introns of the nearby *MCM6* gene. Individuals with sustained expression of lactase carry polymorphisms that function as strong distal enhancers for *LCT* gene expression, thus compensating the normal down regulation of *LCT* transcription during weaning, and consequently sustaining lactase expression in adulthood^3^. Enhancer polymorphisms have been suggested to have been positively selected following the domestication of cattle and camels in the Middle East over five thousand years ago^9^,^10^.

The symptoms resulting from ATH can be managed by reducing lactose intake, for example by removing dairy products from the diet. An alternative approach for ATH, and the approach of choice for CLD, is the use of lactase supplements, widely available from pharmacies. These supplements provide lactase isolated from a variety of sources, including yeast and bacteria, in a liquid or pill-based form that can be taken with or added to lactose containing food. The supplement will hydrolyze a proportion of the lactose present in the food to glucose and galactose products, thus permitting their absorption and preventing accumulation of undigested lactose substrate in the gut.

Based upon the use of lactase supplements as a dietary aid, we have developed a simple enzymology laboratory experiment suitable for first year biomedical science or pharmacy students. This laboratory experiment takes advantage of commercially available lactase supplements, and uses ortho-nitrophenol-*beta*-D-galactopyranoside (ONPG) to provide a colorimetric end point for measuring cleavage of glycosidic bonds by lactase (**Figure 3**)^11^. ONPG acts an artificial substrate for lactase, which when subjected to hydrolysis by this enzyme produces D-galactose and ortho-nitrophenol. The latter product has a yellow color, absorbing light at a wavelength of 420 nm. By quantifying any changes in absorbance at 420 nm following the exposure of ONPG to lactase, it is possible to estimate the activity of this enzyme. This laboratory experiment provides a demonstration of enzymatic hydrolase activity. By building in additional replicates and carrying out assays in different conditions, it is possible to incorporate more sophisticated analyses of enzyme kinetics, providing a valuable real-life example of enzymes in action relevant to human health.

## Protocol

NOTE: The protocol below was developed to be held over the course of two hours (including completion of in-class worksheets) in a purpose-built teaching laboratory. The experimental steps were deliberately put together to exclude more detailed analysis of enzyme kinetics (carried out in the context of this course using model data); however — as noted in the discussion — the protocol provides a template for more advanced analyses depending upon time, facilities and student attainment level.

SAFETY: This practical should be carried out according to the rules of Good Chemical Laboratory Practice (GCLP) — personal protective equipment, including fastened laboratory coat, disposable gloves and safety glasses should be worn at all times in the laboratory.

### 1. Extracting the Enzyme

1. Crush one lactase tablet, containing 200 mg of enzyme, to an even powder using a mortar and pestle.
2. Place the resulting powder into a 15 mL tube labelled "suspension" and resuspend in 10 mL of 100 mM phosphate buffered saline (PBS).
3. Vortex for 1 min to maximize enzyme extraction.
4. Transfer 1 mL from the suspension into a 1.5 mL centrifuge tube and centrifuge for 1 min at 10,000 x *g *to sediment solid particles.
5. Transfer 500 µL of the supernatant to a clean 1.5 mL tube labelled "Lactase Extract".

### 2. Observing the Colorimetric Reaction

1. Place 390 µL of 100 mM PBS into a 1.5 mL tube labelled "Reaction A".
2. Add 100 µL of 5 mM ONPG solution and mix well by vortexing. CAUTION: This practical uses ortho-Nitrophenol-*beta*-D-galactopyranoside (ONPG) as a substitute for lactose. Since ONPG is a phenolic compound, it should be handled with caution. In the event of skin contact with ONPG, the exposed skin should be washed immediately. Any spills should be wiped up immediately with paper towels to be disposed of into the appropriate waste stream.
3. Add 10 µL of extract into the reaction A tube and mix well by vortexing.
4. Observe the reaction mixture for 5 minutes and note any colorimetric changes to the solution.

### 3. Measuring Enzyme Activity

1. Set up two 1.5 mL tubes: one labelled "Reaction B", one labelled "Control".
2. Add 390 µL of 100 mM PBS to the reaction B tube, 400 µL of 100 mM PBS to the control tube and then add 100 µL of 5 mM ONPG to each tube.
3. Mix the contents by vortexing.
4. Add 10 µL of lactase extract to the reaction B tube, mix by vortexing and allow the reaction to proceed for 1 min at room temperature.
5. Once 1 min has elapsed, add 500 µL of 1 M sodium carbonate to both tubes to inhibit the lactase enzyme by increasing the pH, thereby terminating the reaction.
6. Transfer 500 µL from each tube into clean spectrophotometer cuvettes and measure absorbance at 420 nm using the spectrophotometer.
7. Note absorbance for reaction B and control samples, and then subtract the control value from the reaction value to derive the change in absorbance due to hydrolysis of ONPG in the presence of active enzyme.

### 4. Effect of Temperature on Enzyme Activity

1. Set up three control tubes and three reaction tubes as described in section 3 and label these****"4 °C", "Room Temp", and "37 °C".
2. Following addition of enzyme extract, incubate one tube on ice, one at room temperature, and one at 37 °C (in a pre-heated water bath).
3. Allow the reactions to proceed for 1 min and then terminate the reaction by adding 500 µL of 1 M sodium carbonate to each tube.
4. Measure the absorbance at 420 nm for each tube as described in section 3. Record the values, subtracting the control value from the reaction value in each case.

## Representative Results

Representative results for Section 2 of the protocol are shown in Figure 4A. The control reaction, in the absence of lactase enzyme, remains clear whilst the reaction solution, containing extract from the lactase tablet, turns yellow as ONPG is hydrolyzed releasing *ortho*-nitrophenol. Figure 4B shows quantification using arbitrary units from Section 4 of the protocol, following analysis of samples using the spectrophotometer. Production of ortho-nitrophenol is lowest when the reaction is incubated on ice, and highest when the reaction is carried out at 37 °C.

**Figure:**
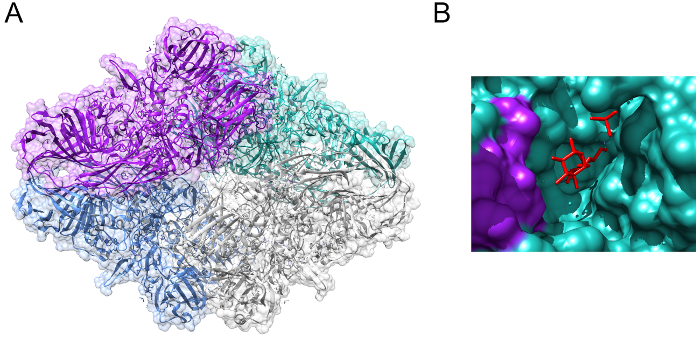

Figure 1**:***** E. coli *****beta-galactosidase. **(**A**) Crystal structure of beta-galactosidase from *Escherichia coli*, showing the tetrameric structure of the active complex. (**B**) Allolactose (shown in red) bound to the active site of beta-galactosidase. Images generated from PDB 1JZ8^4^. Please click here to view a larger version of this figure.

**Figure:**
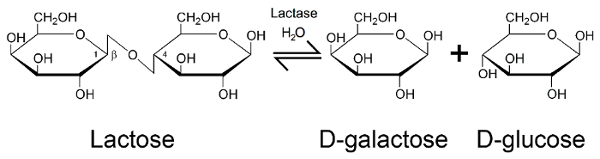

Figure 2**:Enzymatic action of lactase. **Hydrolysis of lactose by lactase to produce beta-D-galactose and beta-D-glucose. Please click here to view a larger version of this figure.

**Figure:**
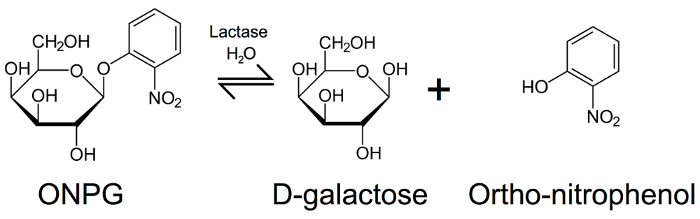

Figure 3**:** **Hydrolysis of ortho-nitrophenol-*****beta*****-D-galactopyranoside. **ONPG hydrolysis by lactase to produce beta-D-galactose and ortho-nitrophenol (which is yellow in color), allowing estimation of lactase activity by measurement of absorbance at 420 nm. Please click here to view a larger version of this figure.

**Figure:**
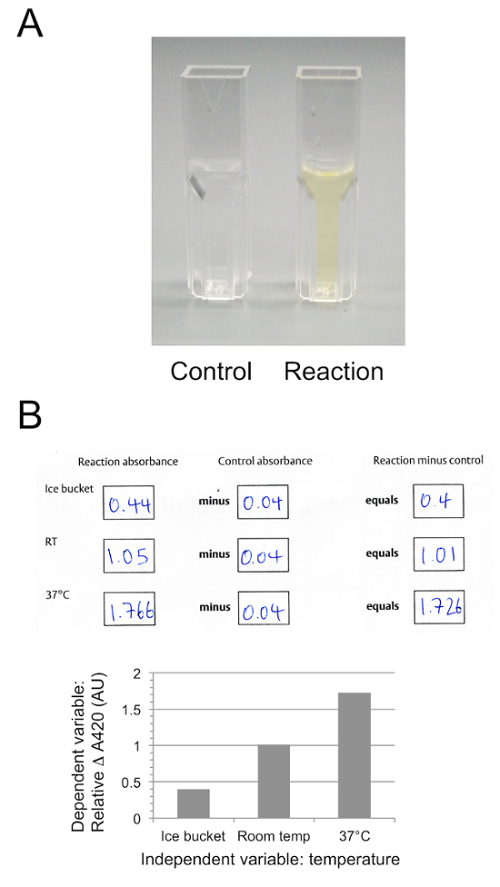

Figure 4**:ONPG hydrolysis. **Representative results of ONPG hydrolysis, showing control and reaction tubes (**A**) and representative data from Section 4 of the protocol recorded on in-class worksheets, showing raw absorbance in arbitrary units at 420 nm, corrected for background and at three different temperatures (**B**). Please click here to view a larger version of this figure.

**Table:** 

**Experimental Condition**	**Experimental variables**
Temperature	0 °C, 10 °C, 20 °C, 30 °C, 50 °C
Substrate concentration	0 mM, 1 mM, 5 mM ,10 mM ONPG
Competitive inhibition	5 mM ONPG plus 0 mM, 1 mM, 5 mM, or 10 mM lactose
Time dependence	0 s, 15 s, 30 s, 1 min, 5 min, 10 min, 20 min
Heat denaturation	Lactase extract heated to 100 °C for 10 min prior to assay

**Table 1: Potential extended experimental condition**. Suggested extended experiments, to be carried out in triplicate, investigating specific aspects of lactase biology.

## Discussion

A detailed knowledge and understanding of enzymes, enzymatic reactions and enzyme kinetics is required for a wide range of topics spanning biology, biomedicine and pharmacology. The protocol described above uses lactase as an example of an enzyme relevant to human health for demonstrating enzymatic reactions, providing key skills that can be used in more advanced laboratory-based experiments.

This protocol has been deliberately designed to be as robust as possible, allowing students with little or no wet laboratory experience to produce interpretable results in a short space of time. In the 2014/2015 academic year at the University of Reading School of Pharmacy, a total of 144 students, organized into groups of 3, carried out the "measuring lactase" experiment, with all groups able to detect some level of enzymatic activity from lactase tablet extracts.

There are a number of critical steps in this protocol. First, it is essential that the initial extraction is efficient, allowing solubilization of enough active enzyme for subsequent analyses. In our experience, first year undergraduates can achieve this by following the protocol as outlined; however some guidance from laboratory demonstrators was required at this point. Although it would seem a self-evident point, a key factor in the success of this protocol is the ability to carefully measure volumes, correctly label tubes and follow instructions. While a certain level of proficiency can be assumed for many students, careful monitoring and a clear request for students to ask for assistance in the event that they are unsure of what to do next provides the greatest likelihood of a successful assay.

Student evaluation can be carried out by the in-class worksheet (available as supplemental material), asking students to note data from the experiments, comment on their results and link to background information/reading from lecture material, or by more detailed, out of class assessment with model data provided allowing for kinetic analyses to be attempted and evaluated.

The protocol presented herein is a simple example of a lactase activity assay under teaching laboratory conditions, and there is ample opportunity to increase the complexity of the experiment to allow more advanced analyses. In particular, the presented protocol provides an introduction to the concept of enzyme kinetics but does not allow for kinetic analysis of experimental data. As such, we would recommend viewing the described protocol as an initial template for classes, with the precise details adapted to suit the specific aims and talents of the student cohort undertaking the experiments. Examples of extended experiments could include: repeated measurements at different temperatures, using different substrate concentrations to allow statistical analysis, kinetic analysis of data (suitable for biochemistry students/majors), starting with several different tablets and allowing the students to evaluate different activity of enzyme in each (with the addition of control tablets, suitable for forensic science students/majors), assessing the impact of heat denaturation, or carrying out competition experiments by addition of lactose or inhibitors of lactase (**Table 1**). A detailed protocol for kinetic analysis of lactase has previously been published^12^.

An important strength of this protocol is that it combines a basic introduction to enzymology and enzyme kinetics with a real-life case study of enzymology in medicine. This makes this laboratory experiment of particular relevance to students of medicine, pharmacy, biomedical sciences and related subjects. Importantly, the fact that there is both a serious medical condition and a more widespread dietary issue associated with lactose catabolism provides the opportunity to raise a wide range of medical and ethical issues with the students. This could include the issues surrounding genetic testing for medical conditions, and associated discussions relating to gene therapy and genetic counselling, as well as the use and sale of medicinal supplements. One major limitation is that the protocol does not allow for precise estimation of enzyme concentration, due to the starting material used for the extraction. A modification to account for this, however, would be to use reagent grade enzyme for the assay. Importantly, this would also allow for more precise calculation of kinetics for the lactase enzyme.

In conclusion, assaying the activity of lactase in a teaching lab environment provides a robust, engaging and interesting introduction to the field of enzyme biology for early stage university students.

## Disclosures

The authors have no conflicts of interest to disclose.
